# Cognitive dysfunctions in multiple sclerosis

**DOI:** 10.1192/j.eurpsy.2026.10348

**Published:** 2026-07-31

**Authors:** V. R. Enătescu

**Affiliations:** Neuroscience, University of Medicine and Pharmacy “Victor Babes” Timisoara, Timisoara, Romania

## Abstract

**Abstract:**

Cognitive dysfunctions cause pervasive and significant disabilities in people with multiple sclerosis. This will increase the global burden of the disease. Different domains of cognition are not equally affected by multiple sclerosis. Among the vulnerable domains of cognition impaired by multiple sclerosis, it should be noted that information processing speed (IPS), verbal memory, visual memory, prospective memory, executive functions, visuospatial processing, language functions, and social cognition are affected. So far, studies have revealed that cognitive dysfunctions in people with multiple sclerosis negatively impact the quality of life, disease management, risk of falls, driving, financial management skills, employment, and attending social and leisure activities. Moreover, their caregiver partners’ health-related quality of life is also diminished. Three batteries are widely used to objectively assess cognition in persons with multiple sclerosis: the Brief Repeatable Battery of Neuropsychological Tests (BRB-N), the Minimal Assessment of Cognitive Function in MS (MACFIMS), and the Brief International Cognitive Assessment for MS (BICAMS). There are a lot of other psychiatric comorbidities or complications of multiple sclerosis that should be identified and treated, such as anxiety, depression, apathy, organic personality disorder, psychoactive substance abuse, and even suicide. All these conditions may worsen the cognitive impairments in people with multiple sclerosis. Some of the psychiatric comorbidities could be a direct consequence of disease-modifying therapy in multiple sclerosis. Whatever the case, the management of this subclinical population of people with multiple sclerosis and cognitive dysfunctions needs specific management, consisting of specific social, psychological, and pharmacological interventions.

**Keywords:**

cognitive dysfunctions, multiple sclerosis, disabilities

**References:**

1. Anonymous, Stern EB. A mind “surrounded by a moat”: a first-person account of cognitive impairment in multiple sclerosis. Cogn Behav Neurol. 2011 Dec;24(4):217-26.

2. Costa SL, Genova HM, DeLuca J, Chiaravalloti ND. Information processing speed in multiple sclerosis: Past, present, and future. Mult Scler. 2017 May;23(6):772-789.

3. De Meo E, Portaccio E, Giorgio A, et al. Identifying the Distinct Cognitive Phenotypes in Multiple Sclerosis. JAMA Neurol. 2021 Apr 1;78(4):414-425.

**Disclosure of Interest:**

None Declared

